# Comparative Analysis of Three Preparation Strategies for AntiMicrobial Peptides and Their Antibacterial Efficacy

**DOI:** 10.3390/cimb48100983

**Published:** 2026-09-24

**Authors:** Meihereayi Mutailifu, Xutong Wang, Maierhaba Sailaijiang, Yuning Zhang, Danni Chen, Kun Xie

**Affiliations:** 1 Key Laboratory of Biological Resources and Ecology of Pamirs Plateau in Xinjiang Uygur Autonomous Region, College of Life and Geographic Sciences, Kashi University, Kashi 844000, China; miray1201@outlook.com (M.M.); wangxutong2002@163.com (X.W.); marhaba0712@163.com (M.S.); zyn20021218@163.com (Y.Z.); 13267822589@163.com (D.C.); 2 College of Biological and Agricultural Sciences, Honghe University, Mengzi 661199, China

**Keywords:** antimicrobial peptides, fusion expression, enterokinase, SNAC chemical cleavage, SUMO protease, antibacterial activity

## Abstract

Antimicrobial peptides (AMPs) are regarded as promising candidates for the post-antibiotic era because of their broad-spectrum bactericidal activity and low propensity to induce bacterial resistance. However, their small molecular weight and intrinsic cytotoxicity toward the expression host result in poor soluble expression and low yields when unmodified AMPs are produced directly in prokaryotic systems. In this study, the coding sequences of three AMPs (Swine_2, P3-3R-8I, and QLX-3DV-1) were assembled by primer-complementary PCR, and nine recombinant expression vectors covering three fusion-tag systems were constructed by restriction digestion and ligation, namely pET32a-Swine_2, pET32a-P3-3R-8I, pET32a-QLX-3DV-1, pET32a-GSHHW-Swine_2, pET32a-GSHHW-P3-3R-8I, pET32a-GSHHW-QLX-3DV-1, pET3C-SUMO-Swine_2, pET3C-SUMO-P3-3R-8I, and pET3C-SUMO-QLX-3DV-1. The resulting plasmids were transformed into Escherichia coli BL21(DE3), and the nine fusion proteins were expressed under IPTG induction and auto-induction. The fusion proteins were purified by Ni-NTA affinity chromatography; the target AMPs were released by enterokinase digestion, SNAC-mediated chemical cleavage, or SUMO protease digestion, and the peptides were recovered by ultrafiltration. The antibacterial activity of the recovered peptides against *E. coli* and Staphylococcus aureus was systematically compared using the agar diffusion assay and the broth microdilution method. All three strategies yielded bioactive AMPs. For a given peptide backbone, the three preparations showed identical MIC values, whereas the peptides released by SUMO protease produced the largest inhibition zones. Because the three preparations of any one backbone share an identical primary sequence and an identical MIC, this difference cannot be attributed to a higher intrinsic potency of the peptide; it is treated here as a semi-quantitative observation. This study provides a complete technical workflow and a methodological reference for the engineered production of AMPs.

## 1. Introduction

Antimicrobial peptides (AMPs) are naturally occurring small-molecule effectors of innate immunity in virtually all living organisms. They possess broad-spectrum bactericidal capacity, a low risk of inducing bacterial resistance, and immunomodulatory functions [1]. The global crisis of antimicrobial resistance has greatly complicated the clinical management of multidrug-resistant bacterial infections, positioning AMPs as one of the most promising alternatives to conventional antibiotics [2]. The optimism surrounding AMPs rests on three mechanistic advantages over conventional antibiotics. First, most conventional antibiotics act on a single molecular target (a specific enzyme, ribosomal subunit, or cell-wall synthase), so a single point mutation or an acquired resistance gene is often sufficient to abolish their activity; AMPs act predominantly on the bacterial cytoplasmic membrane, a target that cannot be extensively re-engineered by the bacterium without losing viability; therefore, resistance generally requires substantial remodelling of the envelope and develops far more slowly [3,4]. Second, AMPs kill rapidly through physical membrane disruption, and many of them retain activity against non-growing or dormant cells and against biofilms, which are refractory to most antibiotics that require active cell division [5,6]. Third, AMPs frequently display additional activities that conventional antibiotics lack, including neutralization of lipopolysaccharide and lipoteichoic acid, modulation of host immune responses, and synergistic potentiation of conventional antibiotics [4,6]. Nevertheless, the large-scale industrial application of AMPs is still restricted by two major bottlenecks: the extremely low yield of natural tissue extraction and the prohibitive cost of solid-phase chemical synthesis. Expression in genetically engineered microorganisms has therefore become the core strategy for the low-cost, large-scale production of AMPs [7].

The antibacterial activity of an AMP is governed by an interdependent set of structural and physicochemical parameters, of which net charge, hydrophobicity, amphipathicity, and conformational flexibility are the most important [8,9]. A net positive charge of roughly +2 to +9 at physiological pH drives the initial electrostatic attraction of the peptide to the anionic surface of the bacterial envelope, which is rich in lipopolysaccharide in Gram-negative bacteria and in teichoic acid and phosphatidylglycerol in Gram-positive bacteria; the zwitterionic and cholesterol-rich membrane of mammalian cells is comparatively neutral and is therefore far less attractive [8,9]. Hydrophobicity, typically expressed as the proportion of nonpolar residues or as the hydrophobic moment, controls the depth to which the peptide inserts into the lipid bilayer and hence the extent of bilayer perturbation [10,11]. Amphipathicity—the spatial segregation of cationic and hydrophobic residues onto opposite faces of an α-helix or a β-sheet—is the key determinant of membrane selectivity because it allows one face of the peptide to remain in contact with the lipid acyl chains while the other engages the anionic head groups [8,11]. Excessive hydrophobicity or an excessively high charge, however, increases the affinity of the peptide for zwitterionic membranes and thus increases hemolytic and cytotoxic side effects; therefore, activity and selectivity must be balanced rather than maximized independently [6,9]. Additional parameters, including peptide length, helicity, and conformational flexibility, further modulate the balance between the carpet, barrel-stave, and toroidal-pore mechanisms of membrane disruption [8,12,13]. Escherichia coli remains the most widely used prokaryotic expression host, yet cationic AMPs encounter two critical barriers. First, AMPs typically have molecular weights of only 2–5 kDa and are therefore highly susceptible to degradation by endogenous host proteases. Second, positively charged AMPs can penetrate the cytoplasmic membrane of *E. coli*, causing host cell death and preventing the efficient production of free AMPs [14]. Toxicity toward the host is a direct consequence of the same mechanism that confers antibacterial activity. The inner membrane of *E. coli* carries a substantial negative surface potential because of its content of phosphatidylglycerol and cardiolipin; therefore, a cationic AMP accumulating in the cytoplasm is electrostatically recruited to the inner face of the membrane, inserts into the bilayer, and dissipates the proton motive force [8,11]. The resulting depolarization halts ATP synthesis, arrests the export and folding of host proteins, and triggers the leakage of ions and metabolites; at higher concentrations, the peptide additionally accumulates in the cytoplasm, where it can bind DNA and RNA and interfere with replication, transcription, and translation [8,12]. Because these effects are exerted on the host itself, the induction of free cationic AMP expression characteristically arrests growth and reduces the viable cell count within a short period. Co-expression with a fusion tag simultaneously improves peptide solubility and mitigates host cytotoxicity because the bulky, generally acidic, or neutral partner masks the cationic character of the AMP and sequesters it in a soluble, inactive form until the tag is removed [15,16]. After expression of the fusion protein, sequence-specific enzymatic or chemical cleavage removes the tag and releases intact, bioactive AMPs, which has become the dominant production pipeline [17,18]. Current tag-removal systems fall into two main categories: protease digestion and sequence-specific chemical cleavage. Enterokinase is a classic, commercially available protease that specifically recognizes the pentapeptide motif DDDDK; it has standardized digestion protocols and is widely used in laboratories [19]. Enterokinase (enteropeptidase, EC 3.4.21.9) is a heterodimeric serine protease of the duodenal brush border; the catalytic light chain recognizes the tetra-aspartate sequence Asp-Asp-Asp-Asp through a cluster of basic residues that form an acidic side-chain binding site, positions the substrate in the catalytic cleft, and hydrolyzes the peptide bond on the carboxyl side of the lysine residue [20]. Because recognition depends almost entirely on the acidic P5–P2 residues and the scissile lysine, enterokinase tolerates a wide range of residues on the amino side of the cleavage site, which allows it to release target peptides with diverse N-termini; the reaction proceeds under mild conditions (pH 7.4–8.0) and is compatible with a variety of buffers [20]. The SUMO fusion tag is recognized by SUMO protease through the intact three-dimensional conformation of SUMO and leaves no residual amino acids after cleavage. This feature maximally restores the native primary structure of the target AMP and makes the system highly compatible with small cationic peptides [16,21,22]. SNAC (sequence-specific nickel-assisted cleavage) is a recently developed enzyme-free technology that mediates peptide bond hydrolysis under mild metal-ion catalysis. It removes the requirement for expensive proteases and substantially reduces raw material costs in scaled-up manufacturing [23]. We selected these three systems for comparison because they represent the three practically available solutions to the tag-removal step and differ in exactly the properties that determine process economics: enterokinase is the established, standardized option with the highest reagent cost; SNAC eliminates the enzyme cost but leaves a residual tetrapeptide; and the SUMO system preserves the native sequence at the expense of an expensive protease [15,16]. No systematic side-by-side comparison of the three on a single set of AMP scaffolds has been reported, and the choice is therefore usually made empirically.

Three cutting-edge pipelines currently drive the development of novel AMPs: remote genomic mining based on protein language models, rational site-directed mutagenesis of native short peptides, and de novo design based on multimodal deep learning. These approaches rapidly generate candidates with low hemolytic activity and robust antibacterial performance [24]. Swine_2 is a remote evolutionary peptide mined from the porcine host and gut microbial genomes by combining the ESM-2 protein language model with the HMD-AMP framework; it belongs to the subset of candidates that share less than 40% sequence identity with known AMPs, and its minimum inhibitory concentration (MIC) against enterotoxigenic *E. coli* (ETEC) and porcine methicillin-resistant *S. aureus* (MRSA) is as low as 1 μM [25]; the values determined here against the two reference strains are higher (Table 4), which is attributable to the different indicator strains, the assay medium and the inoculum used. P3-3R-8I was engineered from an inactive cuticular peptide of Ostrinia nubilalis by the site-specific introduction of arginine and isoleucine residues to modulate net charge and hydrophobicity, forming a stable amphipathic fold that exerts a dual bactericidal effect through membrane permeabilization and inhibition of DNA replication [26]. QLX-3DV-1 was designed by integrating the QLAPD database, AlphaFold2 three-dimensional structure prediction, and M3-CAD multimodal deep learning, and shows potent inhibitory activity against clinical ESKAPE multidrug-resistant pathogens, with MIC values of 4–8 μg/mL [27]. In this study, three representative novel AMPs were used to construct nine recombinant vectors carrying enterokinase, SNAC, or SUMO cleavage sites. The fusion proteins were expressed under both IPTG induction and auto-induction, purified by Ni-NTA chromatography, and processed through three distinct cleavage workflows. We systematically compared peptide recovery, inhibition zone diameters, MIC values, and bacterial viability curves across all treatments, providing comprehensive experimental evidence for the selection of AMP preparation processes tailored to different production scales and activity requirements.

## 2. Materials and Methods

### 2.1. Strains and Plasmids

#### 2.1.1. Expression Host Strains

Chemically competent *E. coli* BL21(DE3) cells were used for recombinant plasmid transformation and fusion protein expression. *E. coli* ATCC 8739 and Staphylococcus aureus ATCC 25923 served as indicator strains for the antibacterial activity assays. The empty pET32a plasmid was maintained in our laboratory.

#### 2.1.2. Recombinant Expression Vectors

Nine recombinant expression vectors were constructed, corresponding to the three AMPs and three cleavage strategies (Table 1). The AMPs recovered after cleavage were designated Swine_2 (DDDDK), P3-3R-8I (DDDDK), QLX-3DV-1 (DDDDK), SHHW-Swine_2, SHHW-P3-3R-8I, SHHW-QLX-3DV-1, Swine_2 (SUMO), P3-3R-8I (SUMO), and QLX-3DV-1 (SUMO).

### 2.2. Gene Synthesis of Amps and Primer Design

#### 2.2.1. Amino Acid Sequences of the Three Amps

The coding sequences of Swine_2, P3-3R-8I, and QLX-3DV-1 were deduced from their amino acid sequences and optimized according to the codon usage preferences of *E. coli*, as described for the heterologous expression of small cationic peptides [3,18].

#### 2.2.2. Primer Design and Gene Amplification

Sequence-specific primers were designed according to the primer-complementary strategy, and PCR was performed to amplify the DNA fragments encoding QLX-3DV-1, P3-3R-8I, Swine_2, GSHHW-QLX-3DV-1, GSHHW-P3-3R-8I, and GSHHW-Swine_2, following the splicing-by-overlap-extension protocol [28]. The tandem SUMO-AMP fragments (SUMO-QLX-3DV-1, SUMO-P3-3R-8I, and SUMO-Swine_2) were amplified using the pET3C-SUMO recombinant plasmid and the individual AMP templates. Detailed primer sequences are listed in Supplementary Table S1.

### 2.3. Construction of Recombinant Expression Vectors

The empty pET3C plasmid and the target gene fragments were double-digested with NdeI/BamHI, whereas the pET32a backbone and the inserts were digested with KpnI/BamHI. The linearized vectors and purified inserts were ligated with T4 DNA ligase, and the ligation mixtures were transformed into competent *E. coli* DH5α cells, essentially as described for standard recombinant AMP vector construction [3,18]. Transformants were spread onto LB agar plates supplemented with 100 μg/mL of ampicillin and incubated overnight at 37 °C. Ten single colonies from each plate were picked and cultured overnight in liquid LB medium containing ampicillin. Colony PCR was performed with universal T7 primers to identify positive clones by using the empty pET32a and pET3C plasmids as negative controls. Nine recombinant plasmids were successfully generated and classified into three groups according to the cleavage motif: the pET32a series carrying the enterokinase DDDDK site, the pET32a-GSHHW series carrying the SNAC cleavage motif, and the pET3C-SUMO series carrying the SUMO protease recognition sequence. The verified positive plasmids were extracted and transformed into competent *E. coli* BL21(DE3) cells for subsequent protein expression.

### 2.4. IPTG-Induced Fusion Protein Expression

Single positive transformant colonies were inoculated into LB medium containing 100 μg/mL of ampicillin and cultured at 37 °C with shaking until the OD600 reached 0.6–0.8, following standard practice for T7 promoter-driven expression [18]. Isopropyl β-D-1- thiogalactopyranoside (IPTG) was added to a final concentration of 0.1 mM, and protein expression was induced for 0–6 h. Cells were harvested at serial time points, resuspended in 1× PBS supplemented with 5× protein loading buffer to a final volume of 100 μL, and analyzed by SDS-PAGE to evaluate the fusion protein yield over the induction period.

### 2.5. Auto-Induction of Fusion Proteins

Auto-induction was performed with the ZYM-5052 system, as originally described by Studier [29] and as subsequently applied to recombinant peptide production [30]. Single colonies carrying the recombinant plasmids were cultured overnight in 4 mL of LB medium supplemented with ampicillin at 37 °C and 220 r/min. The overnight seed cultures were inoculated at a 1% (*v*/*v*) ratio into 10 mL of ZYM5052 auto-induction medium containing 100 μg/mL of ampicillin and incubated at 37 °C and 220 r/min for 16–26 h. Cells were harvested at 16, 18, 20, 22, 24, and 26 h, and SDS-PAGE was used to compare the fusion protein yields obtained with the IPTG induction and auto-induction systems.

### 2.6. Ni-NTA Affinity Chromatography Purification of the Fusion Proteins

Cells harvested after 24 h of auto-induction were used for fusion protein purification with the His60 Ni Gravity Column Purification Kit (Cat. No. 635658, TAKARA, Dalian, China), following the manufacturer’s standard protocol for immobilized metal affinity chromatography [15]. The concentration of the purified fusion proteins was determined with the BCA Protein Assay Kit (Cat. No. P0010, Beyotime, Shanghai, China), and all nine fusion proteins were adjusted to a uniform concentration of 0.1 mg/mL for the subsequent cleavage experiments.

### 2.7. Fusion Protein Cleavage and Amp Recovery Efficiency Calculation

#### 2.7.1. Enterokinase Digestion

One hundred micrograms (1 mL of the normalized fusion protein solution) of each DDDDK-tagged fusion protein was mixed with enterokinase (Cat. No. E8350, Solarbio, Beijing, China) at a volume ratio of 1000:1 and incubated at 25 °C for 16 h, according to the specificity of enterokinase for the Asp-Asp-Asp-Asp-Lys motif [19,20]. After digestion, the mixtures were loaded onto 10 kDa ultrafiltration units to separate the tag fragments and to recover the target AMPs in the filtrate. The recovered peptides were designated Swine_2 (DDDDK), P3-3R-8I (DDDDK), and QLX-3DV-1 (DDDDK). Peptide concentrations were quantified as described in Section 2.7.4, and the recovery rate was calculated using Equations (1)–(3). The molecular weight of the purified AMPs was verified by Tricine SDS-PAGE.

AMP content (μg) = AMP concentration (mg/mL) × final sample volume (mL) × 1000.(1)

Theoretical AMP content (μg) = mass of fusion protein subjected to cleavage (100 μg) × MW(released peptide) ÷ MW(fusion protein).(2)

AMP recovery (%) = [measured AMP content (μg)÷ theoretical AMP content (μg)] × 100%.(3)

#### 2.7.2. SNAC-Mediated Chemical Cleavage

Purified GSHHW-containing fusion proteins (100 μg each) were dissolved in SNAC cleavage buffer (1 mM NiCl_2_, 0.1 M CHES, 0.1 M acetoxime, 0.1 M NaCl, pH 8.6) and incubated at room temperature for 10 h to achieve sequence-specific cleavage at the G↓SHHW motif, as reported by Dang et al. [23]. After cleavage, 10 kDa ultrafiltration was used to remove the high-molecular-weight fusion tags, and the filtrates containing the SHHW-modified AMPs (SHHW-Swine_2, SHHW-P3-3R-8I, and SHHW-QLX-3DV-1) were collected. Peptide quantification was performed as described in Section 2.7.4, the recovery rate was calculated as described in Section 2.7.1, and the peptide size was validated by Tricine SDS-PAGE.

#### 2.7.3. Sumo Protease Digestion

One hundred micrograms of each His-SUMO fusion protein was incubated with SUMO protease at a volume ratio of 1000:1 at 37 °C for 10 h, according to established protocols for SUMO fusion technology [16,21,22]. After digestion, the samples were ultrafiltered through 10 kDa membranes to retain the SUMO tag and to collect the untagged native AMPs (Swine_2 (SUMO), P3-3R-8I (SUMO), and QLX-3DV-1 (SUMO)). Peptide concentrations were determined as described in Section 2.7.4, the recovery rate was calculated as described in Section 2.7.1, and peptide integrity was confirmed by Tricine SDS-PAGE.

#### 2.7.4. Quantification of the Recovered Peptides

Because the colorimetric response of a 2–5 kDa peptide in the BCA assay differs substantially from that of the bovine serum albumin used as the standard, and because the concentrations recovered here (0.03–0.06 mg/mL) lie close to the lower limit of the assay, the BCA assay was used only for the fusion proteins (≥14 kDa, 0.1 mg/mL), for which it is validated, and not for the released peptides. The recovered peptides were quantified by reversed-phase HPLC on a C18 column (4.6 × 150 mm, 5 μm, 300 Å) eluted with a linear gradient of acetonitrile in 0.1% (*v*/*v*) trifluoroacetic acid and calibrated with an external standard series of the corresponding synthetic peptide (5–200 μg/mL, six levels, R^2^ > 0.999;). Every sample was quantified in three independent preparations, and the result was cross-checked by absorbance at 280 nm using the molar extinction coefficients calculated from the sequences with ExPASy ProtParam: ε280 = 4,470 M^−1^ cm^−1^ for Swine_2 and for SHHW-Swine_2, which contain three tyrosine residues, and 16,500 M^−1^ cm^−1^ for P3-3R-8I, QLX-3DV-1 and their SHHW-tagged variants, each of which contains three tryptophan residues; histidine does not contribute appreciably at 280 nm, so the SHHW extension does not change ε280. The final volume of each recovered sample was recorded and is reported in Table 3, together with the molecular weight of the corresponding full-length fusion protein; therefore, every value in the table can be recalculated by the reader. The molecular weight of each full-length fusion protein was obtained as the sum of the vector-encoded tag (15,196 Da for the His-Trx-DDDDK tag of pET32a, 15,801 Da after insertion of the GSHHW motif, and 12,146 Da for the His-SUMO tag of pET3C) and of the inserted peptide; the three tag masses were derived from the vector sequences and are internally consistent with the theoretical yields in Table 3 to within 0.3%.

### 2.8. Antibacterial Activity Evaluation of Recovered AMPs

#### 2.8.1. Agar Diffusion Assay

All purified AMPs and the positive control ampicillin were adjusted to a uniform concentration of 1 μg/μL. Overnight cultures of the indicator strains (*E. coli* ATCC 8739 and *S. aureus* ATCC 25923) were diluted to 1 × 10^6^ CFU/mL and spread evenly onto LB agar plates, following standard agar diffusion practice [31,32]. Wells that were 6 mm in diameter were punched into the solidified agar, and 50 μL of each AMP solution or ampicillin was added to individual wells, with sterile double-distilled water used as the negative control. The plates were incubated at 37 °C for 12–16 h, and the diameter of each inhibition zone was measured with a digital calliper.

#### 2.8.2. Determination of the Minimum Inhibitory Concentration (MIC)

MIC values were determined by the broth microdilution method in cation-adjusted Mueller–Hinton broth, following CLSI standard M07 [33] and the broth dilution procedure described by Wiegand et al. [31]. Serial two-fold dilutions of each AMP were prepared in 96-well plates and mixed with bacterial suspensions at a final concentration of 5 × 10^5^ CFU/mL. The plates were incubated at 37 °C for 16–20 h, and the MIC was defined as the lowest peptide concentration that completely suppressed visible bacterial growth, as confirmed by the absence of turbidity on a microplate reader. Three technical replicates were included for each treatment, and all experiments were independently repeated three times.

#### 2.8.3. Bacterial Viability Assay

Bacterial viability was determined by broth microdilution in 96-well plates as described by Wiegand et al. [31]. Overnight cultures of *E. coli* ATCC 8739 and *S. aureus* ATCC 25923 were diluted to 5 × 10^5^ CFU/mL in cation-adjusted Mueller–Hinton broth, mixed with two-fold serial dilutions of each peptide in a final volume of 100 μL per well, and incubated at 37 °C for 16–20 h without shaking. Growth was recorded as the optical density at 600 nm (OD_600_) on a microplate reader. The inoculated medium without peptide served as the growth control, and sterile medium served as the blank. Relative viability was calculated according to Equation (4):

Relative viability (%) = [(OD_600_, sample − OD_600_, blank) ÷ (OD_600_, growth control − OD_600_, blank)] × 100%. (4)

The peptide concentration that reduced viability to 50% (IC_50_) was obtained by non-linear regression of the viability–concentration curve with a four-parameter logistic model. Each concentration was tested in triplicate, and the assay was independently repeated three times.

### 2.9. Statistical Analysis

All experiments were independently performed at least three times, and all data are presented as means ± standard deviations (SD). The normality of the data was assessed with the Shapiro–Wilk test, and the homogeneity of variance with Levene’s test. Differences in inhibition zone diameter among the three cleavage strategies were analyzed by one-way analysis of variance (ANOVA), followed by Tukey’s honestly significant difference post hoc test; differences in the viability curves among peptide concentrations and preparation strategies were analyzed by two-way ANOVA, followed by Tukey’s multiple-comparison test [GraphPad Prism, version 11.0.2]. Differences were considered statistically significant at *p* < 0.05 and are indicated in the figures as * *p* < 0.05, ** *p* < 0.01, and *** *p* < 0.001. The number of independent replicates is stated in the legend of every panel.

## 3. Results

### 3.1. Physicochemical Characteristics of the Three Candidate AMPs

Three representative AMPs (Swine_2, P3-3R-8I, and QLX-3DV-1) with distinct design origins were selected for this study, and their physicochemical properties are summarized in Table 2. The peptides ranged from 14 to 46 amino acids in length, and all three native AMPs were cationic with positive net charges at physiological pH, fulfilling the core structural requirements for targeting bacterial cytoplasmic membranes. N-terminal fusion of the GSHHW tag consistently increased the net charge and slightly enhanced hydrophobicity: the histidine (H) residues raised the overall positive charge, whereas the tryptophan (W) residue increased hydrophobicity, enabling the directional optimization of AMP function.

Net charge was calculated as (Lys + Arg + His) − (Asp + Glu) at pH 7.0, and hydrophobicity as the grand average of hydropathicity (GRAVY) index; both were computed from the sequences with ExPASy ProtParam.

### 3.2. PCR Amplification of the AMP Coding Sequences

Primer-complementary PCR successfully amplified nine target DNA fragments, including the native AMP genes, GSHHW-tagged AMP sequences, and SUMO-AMP tandem fragments. Agarose gel electrophoresis (3%) showed distinct specific bands of the expected molecular size, confirming the full-length synthesis of all nine target gene fragments (Figure 1). We note that lanes 4 and 5 (P3-3R-8I and GSHHW-P3-3R-8I) and lanes 7 and 8 (QLX-3DV-1 and GSHHW-QLX-3DV-1) migrate very close to one another. This is expected: the GSHHW tag adds only five codons, i.e., 15 bp, and on a 3% agarose gel, a 15 bp difference between fragments of 63–78 bp cannot be resolved. The identity of these fragments was therefore not judged from their mobility but confirmed by Sanger sequencing of the corresponding recombinant plasmids (Section 3.3), which showed 100% identity with the designed sequences. The SUMO-containing fragments (lanes 3, 6, and 9) are clearly larger, as expected from the addition of the ~330 bp SUMO coding sequence.

### 3.3. Construction and Molecular Validation of the Recombinant Vectors

Nine recombinant plasmids were generated by inserting the nine target fragments into the pET32a and pET3C backbones. Colony PCR with universal T7 primers yielded insert-containing bands consistent with the theoretical molecular weights for all positive transformants (Figure 2). Sanger sequencing confirmed 100% sequence identity between all inserted fragments and the designed sequences, with no point mutations, deletions, or insertions detected.

### 3.4. Comparison of Fusion Protein Expression Under IPTG Induction and Auto-Induction

All nine recombinant plasmids were transformed into *E. coli* BL21(DE3), and fusion protein expression was induced by IPTG or by auto-induction. SDS-PAGE analysis confirmed the successful expression of all nine fusion proteins under both induction strategies (Figure 3). Notably, auto-induction yielded markedly higher fusion protein titers than short-term IPTG induction. Therefore, cells harvested after 24 h of auto-induction were used for the subsequent purification workflow.

### 3.5. Soluble Expression and Ni-NTA Purification of the Fusion Proteins

Cells collected after 24 h of auto-induction were lysed by sonication, and the majority of the fusion proteins were detected in the soluble supernatant fraction. Ni-NTA gravity affinity chromatography achieved high-purity enrichment of all His-tagged fusion proteins, providing sufficient substrate for the downstream cleavage assays (Figure 4A–C).

### 3.6. Efficiency of the Three Cleavage Strategies and AMP Recovery Yields

Enterokinase digestion, SNAC chemical cleavage, and SUMO protease digestion all efficiently released the target AMPs from the fusion backbones. Tricine SDS-PAGE revealed discrete bands corresponding to the theoretical molecular weights of each AMP variant, and ultrafiltration yielded highly purified single-component AMP preparations (Figure 4A–C). The fusion proteins (≥14 kDa) were quantified with the BCA assay, for which the assay is validated, whereas the recovered peptides (2–6 kDa) were quantified by reversed-phase HPLC against synthetic peptide standards, with A280 as an orthogonal check (Section 2.7.4, Figure 4D–E). Because the target peptide accounts for only 11–29% of the mass of each fusion protein, the recovery was normalized to the theoretical maximum yield of the released peptide rather than to the total mass of the input fusion protein (Equations (1)–(3), Section 2.7.1). Table 3 now reports, for each of the nine constructs, the molecular weight of the full-length fusion protein, the molecular weight of the released peptide, the theoretical maximum yield obtainable from 100 μg of fusion protein, the final sample volume, and the measured peptide mass; therefore, every recovery value can be recalculated independently. The measured masses obtained with the BCA assay in the previous version of this manuscript have been removed: they exceeded the theoretical maximum yield for every construct (apparent recoveries of 140–381%) and are therefore not interpretable. The re-quantified masses and the corresponding recovery rates are reported in Table 3. Because these values were obtained with a method validated for 2–6 kDa peptides, they provide a reliable basis for comparing the three cleavage strategies.

The molecular weight of each full-length fusion protein was calculated as the sum of the vector-encoded tag (15,196 Da for the His-Trx-DDDDK tag of pET32a; 15,801 Da after insertion of the GSHHW motif; 12,146 Da for the His-SUMO tag of pET3C) and the inserted peptide.

Data are means ± SD of three independent preparations, determined by RP-HPLC against the corresponding synthetic peptide and cross-checked by A280 (Section 2.7.4).

Recovery (%) = (measured peptide mass/theoretical maximum yield) × 100% (Equation (3)).

The BCA-derived masses reported in the previous version of this table (35–55 µg, i.e., apparent recoveries of 140–381%) have been removed because the BCA assay is not valid for peptides of 2–6 kDa.

### 3.7. Antibacterial Activity of the AMPs Generated by the Three Cleavage Approaches

Agar diffusion assays showed that all nine AMP variants exhibited measurable inhibitory activity against both *E. coli* ATCC 8739 and *S. aureus* ATCC 25923 (Figure 5A,A′,B,B′). For a given AMP backbone, the peptides released by SUMO protease digestion produced significantly larger inhibition zones than the corresponding SNAC-modified and enterokinase-digested peptides (one-way ANOVA with Tukey’s post hoc test, *p* < 0.05), and the SNAC-derived peptides, in turn, produced larger zones than the enterokinase-derived peptides. Among the three native scaffolds, QLX-3DV-1 displayed the strongest overall antibacterial potency, with the largest inhibition zones against both indicator strains. Broth microdilution gave MIC values that were identical for the enterokinase-, SNAC-, and SUMO-derived preparations of each backbone (Table 4). This identity is the expected result for the enterokinase and SUMO preparations, which release the same native sequence, and it shows that the residual SHHW tetrapeptide does not change the MIC determined in liquid medium. We therefore no longer interpret the larger inhibition zones of the SUMO preparations as evidence of a higher recovery. Because every sample was applied at the same nominal concentration (1 μg/μL) and volume (50 μL), and because the MIC values are identical, equal amounts of active peptide would be expected to give zones of comparable size; the observed differences must therefore arise from factors other than intrinsic potency. Three such factors can be identified: (i) The concentration assigned to each preparation was derived from the quantification step; therefore, any systematic error in that step propagates directly into the amount of peptide applied per well. (ii) The agar diffusion assay is diffusion-dependent; therefore, the zone diameter is sensitive to the aggregation state of the peptide and to low-molecular-weight residues carried over from the cleavage and ultrafiltration buffers (Ni^2+^, CHES, acetone oxime, and imidazole), which differ between the three workflows and are not removed by a 10 kDa ultrafiltration step. (iii) The SNAC-derived peptides carry the N-terminal SHHW extension, which lowers their net charge-to-mass ratio and their diffusion rate. Distinguishing among these contributions requires the preparations to be re-quantified by a method validated for 2–5 kDa peptides and the assay to be repeated at equal confirmed active-peptide concentrations; until then, the inhibition-zone data are treated as a semi-quantitative ranking and are not used to support any claim about relative recovery (Section 4). The viability curves (Figure 5C–K) showed a concentration-dependent decrease in relative viability for all nine preparations and displayed the same potency order as the MIC data, confirming that the three cleavage strategies deliver peptides of comparable intrinsic activity.

MIC values were determined by broth microdilution in cation-adjusted Mueller–Hinton broth (CLSI M07) in three independent experiments, each performed in triplicate; molar concentrations were calculated from the molecular weights given in the table. Ampicillin was included as a positive control and sterile water as a negative control on every plate.

## 4. Discussion

In this study, primer-complementary PCR enabled the rapid assembly of three novel AMP coding sequences and the construction of nine distinct prokaryotic recombinant vectors representing three cleavage-tag systems. We systematically compared the peptide recovery efficiency and the residual antibacterial activity obtained with enterokinase digestion, SNAC chemical cleavage, and SUMO protease digestion, thereby establishing comprehensive experimental evidence to guide the selection of AMP production processes for different application scenarios.

Primer-complementary PCR eliminates the requirement for native genomic templates and relies solely on the annealing and extension of custom oligonucleotide primers to assemble short peptide coding sequences. This technique is simple to perform and shortens the vector construction timeline, making it ideal for AMPs of fewer than 50 amino acid residues [28]. Codon optimization combined with the Trx and SUMO solubility tags alleviates the cytotoxicity of cationic AMPs toward *E. coli* host cells and significantly increases the yield of soluble fusion protein [34]. Auto-induction media exploit the metabolic regulation of carbon sources to autonomously activate T7 promoter-driven expression, achieving higher cell density and total fusion protein output than short-term IPTG induction. This property makes auto-induction superior for laboratory-scale batch purification and for industrial scale-up [30]. Enterokinase is the traditional gold-standard reagent for tag removal and is ubiquitous in routine laboratory workflows for fusion protein construction. Its appeal lies in the exquisite specificity of the light-chain catalytic domain for the Asp-Asp-Asp-AspLys motif, which allows the target peptide to be released under mild conditions and with an authentic N-terminus [20]. However, the high market price of commercial enterokinase greatly increases the raw material expenditure of large-scale industrial manufacturing, and the long incubation (16 h at 25 °C) increases the risk of non-specific degradation [15,35]. The enzyme-free SNAC nickel-mediated chemical cleavage strategy relies on low-cost buffer salts and nickel ions to mediate sequence-specific peptide bond hydrolysis, giving it a clear economic advantage for mass production [23]. Nevertheless, SNAC cleavage leaves a four-amino-acid SHHW tail at the N-terminus of the AMP, which subtly alters the net charge and hydrophobic balance. Because AMP activity is governed by precisely these parameters [8,9], even a short residual tag can perturb the electrostatic recruitment to the bacterial surface and the depth of insertion into the bilayer, which plausibly accounts for the partial attenuation of activity observed here [10,11]. SUMO protease recognizes the intact three-dimensional fold of SUMO rather than a linear amino acid motif and catalyzes cleavage without introducing any residual amino acids [16,22]. This feature fully preserves the native primary sequence and the conformational characteristics of the AMP, maximally retaining its intrinsic bactericidal activity. SUMO digestion also gives a moderately higher peptide recovery than the other two strategies, although the high cost of commercial SUMO protease restricts its application to small-batch preparation where stringent preservation of activity is required [16,36]. Modern AMP discovery pipelines have shifted from crude biological extraction to computer-aided rational design, encompassing three dominant frameworks: remote genomic mining, site-directed rational mutagenesis, and de novo design based on multimodal deep learning [37,38,39,40,41]. Swine_2 was identified by mining the porcine genome with the ESM-2 protein language model for evolutionarily distant peptide scaffolds, overcoming the limitations of traditional homology-based screening and exerting potent inhibitory effects against porcine multidrug-resistant pathogens [25]. P3-3R-8I was engineered by targeted amino acid substitution to fine-tune charge and hydrophobicity, establishing a dual bactericidal mechanism of membrane permeabilization and intracellular DNA interference that reduces the likelihood of single-target resistance mutations [26]. QLX-3DV-1 integrates protein sequence data, AlphaFold2 structural prediction, and multi-dimensional feature learning to design broad-spectrum AMPs that are active against clinical ESKAPE multidrug-resistant strains [27]. The diversified bactericidal modes of these three AMPs circumvent the drawbacks of conventional membrane-lytic peptides that readily induce bacterial resistance, positioning them as leading candidate molecules for next-generation anti-infective therapeutics [25,26,27]. The pET32a backbone intrinsically carries the Trx and His solubility tags together with the canonical DDDDK enterokinase recognition site, which simplifies vector manipulation. In this study, insertion of the GSHHW sequence upstream of the enterokinase motif introduced SNAC cleavage capability into the same plasmid scaffold, reducing redundant vector construction work. The pET3C-SUMO vector is purpose-built for tag-free peptide production, and the SUMO fusion tag outperforms the Trx tag in improving the solubility of small cationic AMPs, consistent with previously published findings [42]. Each cleavage strategy has distinct advantages and limitations, allowing flexible selection according to manufacturing scale, cost constraints, and antibacterial activity thresholds: SUMO protease digestion is preferred for high-activity, small-volume sample screening; SNAC chemical cleavage is optimal for cost-sensitive industrial mass production; and standardized enterokinase digestion remains a convenient preliminary screening tool for routine laboratory workflows.

All three fusion-cleavage pipelines share a common limitation: a substantial fraction of the target peptide is lost during processing. Four mechanisms contribute to this loss, and each can be addressed experimentally. First, peptides of 2–5 kDa are close to the nominal molecular-weight cutoff of the 10 kDa ultrafiltration membrane, and because the separation of an ultrafiltration membrane is a distribution rather than a step function, a fraction of the peptide is co-retained with the fusion tag or passes slowly enough to be lost in the retentate [15]. Using a membrane with a lower cutoff, or a two-stage separation in which the retentate is re-diluted and re-filtered, would reduce this loss. Second, cationic peptides adsorb non-specifically to the polyethersulfone membrane and to the surfaces of tubes and filter units through a combination of electrostatic and hydrophobic interactions; a systematic loss of this kind is concentration-dependent and is most severe at the low concentrations used here, so increasing the peptide concentration, adding a low concentration of a non-ionic surfactant, or using low-binding consumables would mitigate it [15]. Third, the composition of the cleavage buffer may promote aggregation of the released peptide, particularly for the more hydrophobic variants, and aggregates are efficiently retained by the ultrafiltration membrane; buffer composition, ionic strength, and pH should therefore be optimized for each peptide. Fourth, incomplete cleavage of the fusion protein leaves the target peptide covalently attached to the tag and therefore retained, which is why the recovery follows the same order as the cleavage efficiency observed on Tricine SDS-PAGE [16]. Beyond recovery, the choice of a cleavage system for industrial production is an economic optimization rather than a purely technical one. The relevant quantity is the cost per unit of active peptide, i.e., the sum of the reagent cost per batch and the amortized cost of labour and consumables divided by the mass of active peptide obtained, which is the product of the cleavage yield and the recovery [15]. This quantity can be estimated from the data reported here: enterokinase and SUMO protease contribute a reagent cost that is essentially fixed per batch of fusion protein, whereas the SNAC buffer salts are negligible in comparison; therefore, the break-even point is set by the ratio of the reagent cost to the difference in recovered active peptide. Applying this framework to our data, SNAC cleavage becomes the most economical option once the batch size exceeds the point at which the saved protease cost outweighs the penalty of the lower recovery and the residual SHHW tag, whereas SUMO protease remains preferable where the native sequence and maximal specific activity are required and the batch volume is small. We have deliberately restricted the claim to this qualitative break-even argument and have not quoted absolute costs because reagent prices vary considerably between suppliers and regions; a formal techno-economic analysis would require the actual quotations and the labour cost for each step and is beyond the scope of the present study. Collectively, this study demonstrates that all three cleavage systems reliably generate bioactive Swine_2, P3-3R-8I, and QLX-3DV-1 variants, and quantitatively delineates the gradients in recovery efficiency, residual antibacterial activity, and production costs among SUMO protease digestion, SNAC chemical cleavage, and enterokinase digestion. Importantly, the three preparations of each backbone gave identical MIC values; therefore, the differences observed in the agar diffusion assay reflect the amount of active peptide delivered rather than a difference in intrinsic potency—this distinction should be borne in mind when the three strategies are compared. The standardized experimental workflow and methodological comparison presented here provide a critical reference for the large-scale engineered production of AMPs obtained by protein language model mining, rational mutagenesis, and deep-learning-based de novo design, thereby advancing the industrial translation of AMPs as alternatives to traditional antibiotics [43]. Two core bottlenecks persist in the industrial recombinant production of AMPs: prohibitive manufacturing costs and unstable in vitro antibacterial activity. Subsequent research could integrate immobilized biocatalysis and continuous ultrafiltration to refine the entire production pipeline [44]. Site-directed modification of cleavage sites guided by AlphaFold2 structural prediction represents a viable strategy to mitigate the activity attenuation caused by residual N-terminal amino acids [45]. From an industrial economic perspective, SNAC chemical cleavage delivers superior cost-effectiveness for large-scale manufacturing [46]. Iterative improvements in protein language models will expand the pool of low-cytotoxicity remote AMP scaffolds and broaden the available production feedstock [47]. Coupled SUMO–SNAC dual cleavage workflows are expected to become the main optimization direction for pilot-scale production, balancing antibacterial activity against manufacturing expenditure [46,48].

## 5. Conclusions

Primer-complementary PCR successfully amplified the coding sequences of the three AMPs (Swine_2, P3-3R-8I, and QLX-3DV-1). Restriction digestion and T4 ligase-mediated cloning generated nine recombinant expression vectors divided into three series: pET32a (enterokinase cleavage), pET32a-GSHHW (SNAC chemical cleavage), and pET3C-SUMO (SUMO protease cleavage).

All three preparation strategies yielded AMPs with intact antibacterial activity. SUMO protease digestion preserves the native peptide sequence intact and, in the present data set, gave the largest inhibition zones; SNAC chemical cleavage provides the most cost-effective and operationally simple workflow for industrial scale-up; and enterokinase digestion remains the most mature and widely adopted standard method for the routine laboratory preparation of bioactive peptides. The three preparations of each peptide backbone showed identical MIC values, indicating that the choice of cleavage strategy affects the amount of active peptide obtained rather than the intrinsic potency of the peptide. Based on the re-quantified data in Table 3, SUMO protease digestion gave the highest peptide recovery, followed by SNAC chemical cleavage and enterokinase digestion.

## Figures and Tables

**Figure 1:**
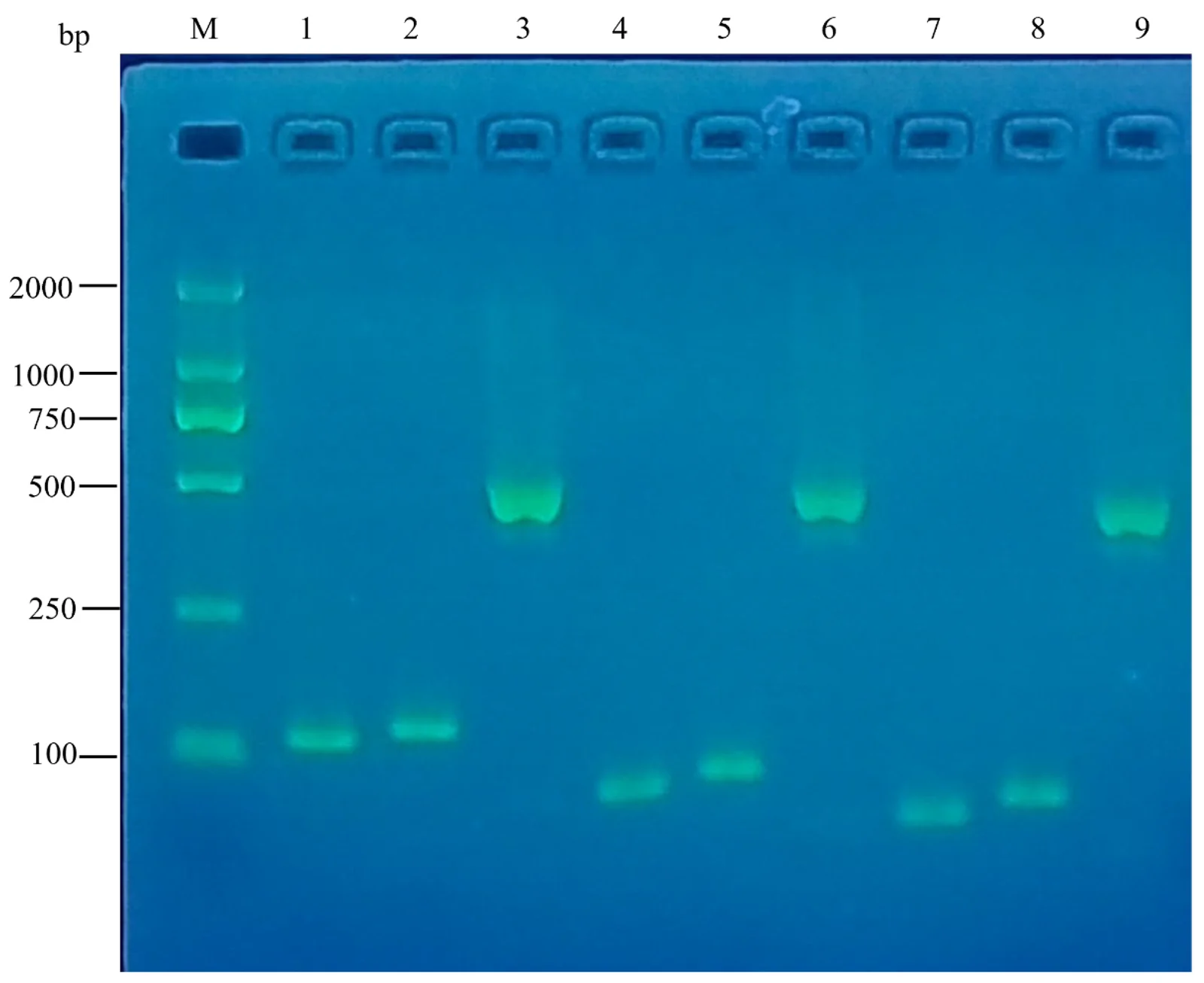
PCR amplification of the AMP genes on a 3% agarose gel. M: DL2000 DNA marker; lane 1: Swine_2 (123 bp); lane 2: GSHHW-Swine_2 (138 bp); lane 3: SUMO-GSHHW-Swine_2 (~468 bp); lane 4: P3-3R-8I (63 bp); lane 5: GSHHW-P3-3R-8I (78 bp); lane 6: SUMO-GSHHW-P3-3R-8I (~408 bp); lane 7: QLX-3DV-1 (42 bp); lane 8: GSHHW-QLX-3DV-1 (57 bp); lane 9: SUMO-GSHHW-QLX-3DV-1 (~387 bp). Lanes 4 and 5 and lanes 7 and 8 differ by only 15 bp and are therefore not resolved on this gel; their identity was confirmed by Sanger sequencing (Section 3.3).

**Figure 2:**
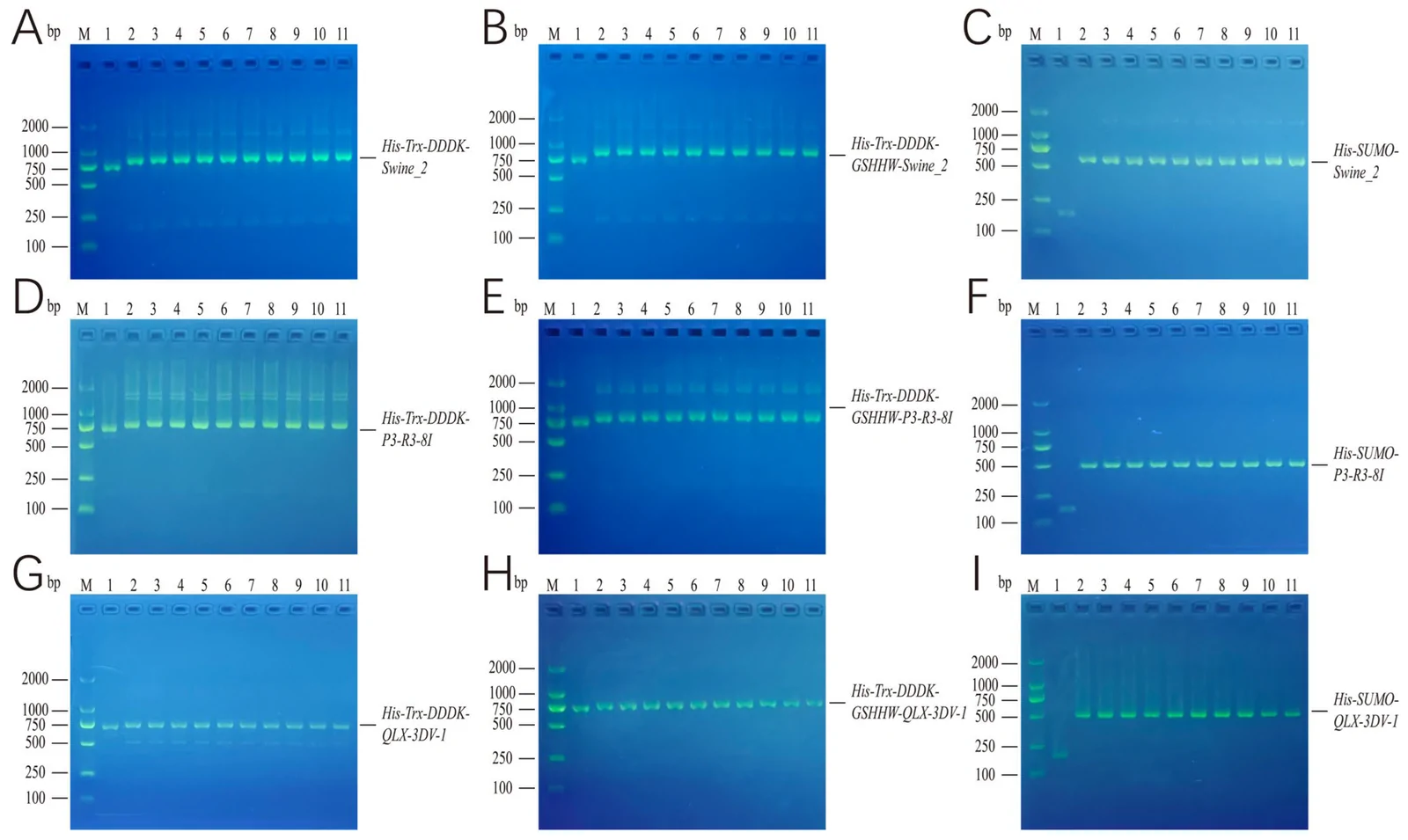
Identification of positive clones by colony PCR. Ten randomly selected single colonies grown on LB plates containing ampicillin were cultured overnight in liquid LB medium containing ampicillin, and colony PCR was performed with universal T7 primers. The panels show the results for transformants carrying pET32a-Swine_2 (**A**), pET32a-GSHHW-Swine_2 (**B**), pET3C-SUMO-Swine_2 (**C**), pET32a-P3-3R-8I (**D**), pET32a-GSHHW-P3-3R-8I (**E**), pET3C-SUMO-P3-3R-8I (**F**), pET32a-QLX-3DV-1 (**G**), pET32a-GSHHW-QLX-3DV-1 (**H**), and pET3C-SUMO-QLX-3DV-1 (**I**). In panels (**A**,**B**,**D**,**E**,**G**,**H**), lane 1 is the colony PCR result for cells carrying the empty pET32a plasmid, and lanes 2–11 are randomly selected colonies carrying the corresponding recombinant plasmid. In panels (**C**,**F**,**I**), lane 1 is the result for cells carrying the empty pET3C-SUMO plasmid, and lanes 2–11 are randomly selected colonies carrying the corresponding recombinant plasmid.

**Figure 3:**
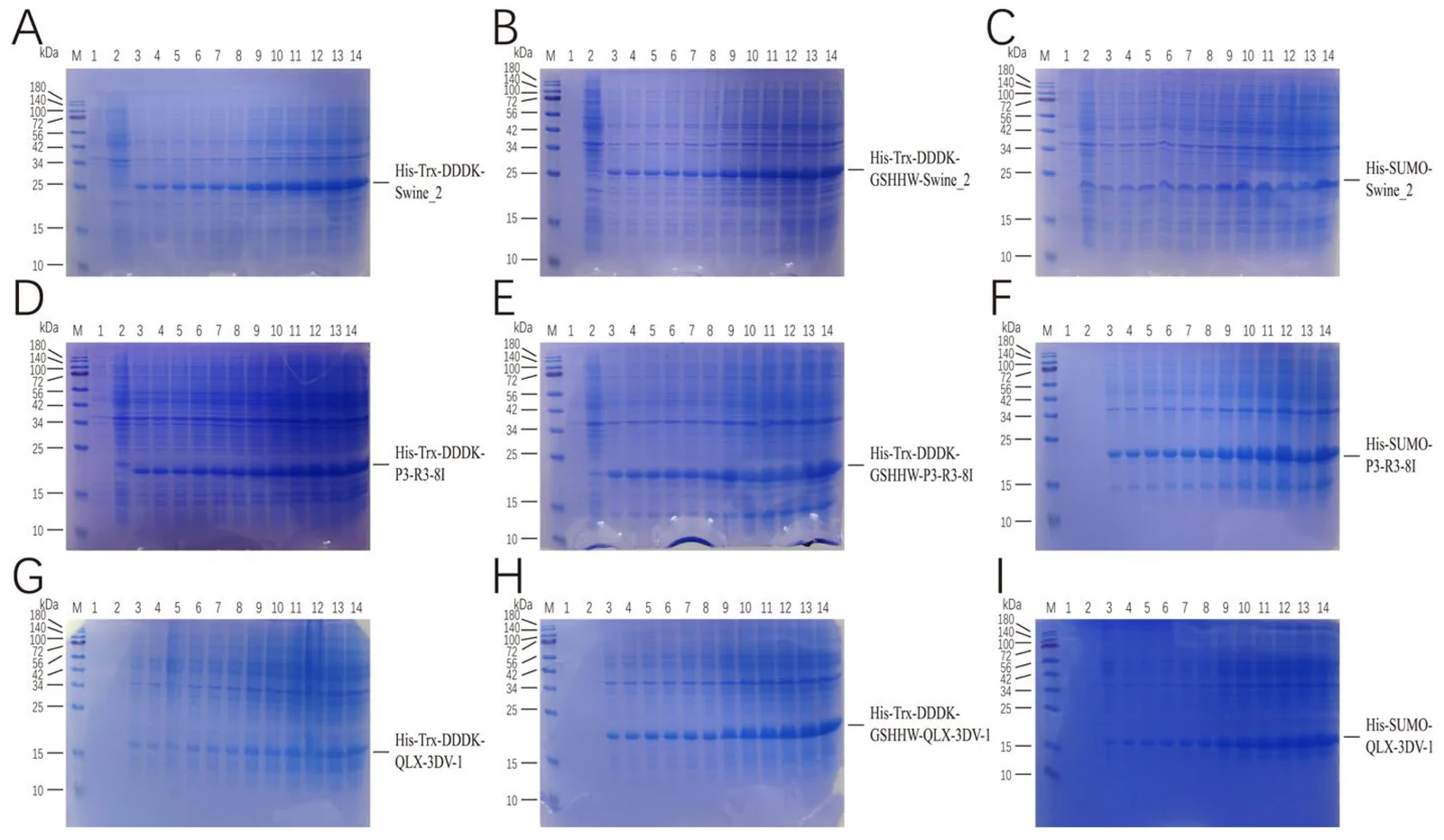
SDS-PAGE analysis of fusion protein expression. Expression was induced with 0.1 mM IPTG or by auto-induction medium. Cells were harvested at 0–6 h after IPTG induction and at 16, 18, 20, 22, 24, and 26 h after auto-induction, and 15% SDS-PAGE was used to detect the expression of His-Trx-DDDDK-Swine_2 (**A**), His-Trx-DDDDK-GSHHW-Swine_2 (**B**), His-SUMOSwine_2 (**C**), His-Trx-DDDDK-P3-3R-8I (**D**), His-Trx-DDDDK-GSHHW-P3-3R-8I (**E**), HisSUMO-P3-3R-8I (**F**), His-Trx-DDDDK-QLX-3DV-1 (**G**), His-Trx-DDDDK-GSHHW-QLX-3DV-1 (**H**), and His-SUMO-QLX-3DV-1 (**I**). M: protein marker; lane 1: cells carrying the empty pET32a plasmid (A, B, D, E, G, and H) or the empty pET3C plasmid (**C**,**F**,**I**); lanes 2–8: expression at 0–6 h after IPTG induction; lanes 9–14: expression at 16, 18, 20, 22, 24, and 26 h after auto-induction. All nine panels (**A**–**I**) follow the same layout.

**Figure 4:**
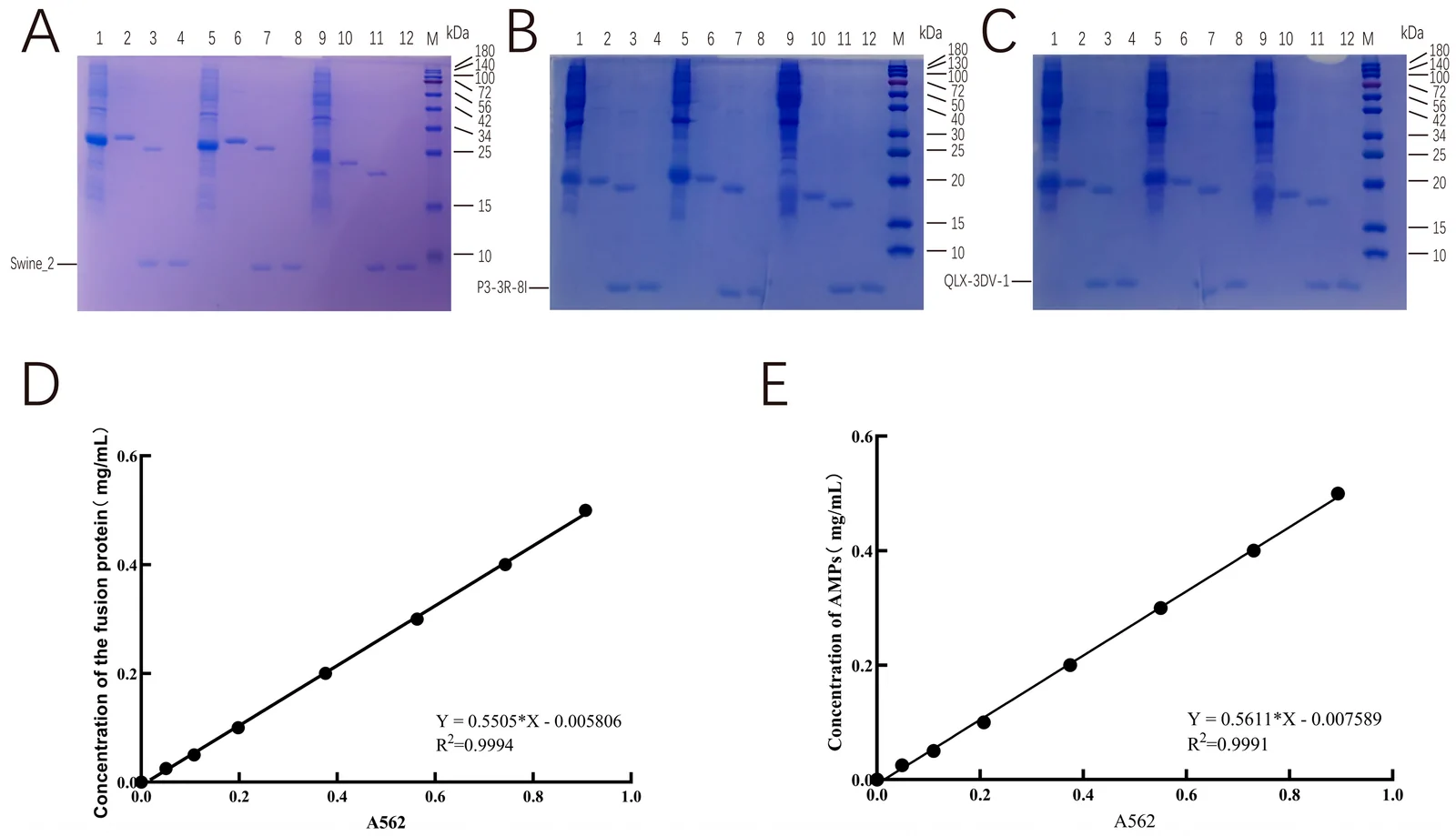
Solubility, cleavage efficiency, and protein quantification of the fusion proteins. Tricine SDS-PAGE analysis of the soluble expression of the fusion proteins in the supernatant and of the cleavage efficiency of enterokinase, SNAC chemical cleavage, and SUMO protease. Quantification was performed for the fusion proteins before cleavage and for the AMPs recovered after cleavage. Panels (**A**–**C**) show, respectively, the results for the Swine_2, P3-3R-8I, and QLX-3DV-1 series; panel (**D**) shows the BCA standard curve used for the fusion proteins (OD562, 0–1000 μg/mL, R^2^ > 0.999); and panel (**E**) the RP-HPLC standard curve used for the recovered AMPs (peak area versus concentration of the corresponding synthetic peptide, 5–200 μg/mL, six levels, R^2^ > 0.999). The BCA assay was not used for the released peptides (Section 2.7.4). Within each of panels (**A**–**C**): (1) expression of the His-Trx-DDDDK-AMP fusion after 24 h of auto-induction; (2) fusion protein in the supernatant after Ni-NTA purification; (3) enterokinase cleavage of the purified fusion protein; (4) ultrafiltration-concentrated AMP released by enterokinase; (5) expression of the His-Trx-DDDDK-GSHHW-AMP fusion after auto-induction; (6) fusion protein in the supernatant after Ni-NTA purification; (7) SNAC chemical cleavage of the purified fusion protein; (8) ultrafiltration-concentrated SHHW-AMP; (9) expression of the His-SUMO-AMP fusion after auto-induction; (10) fusion protein in the supernatant after Ni-NTA purification; (11) SUMO protease cleavage of the purified fusion protein; (12) ultrafiltration-concentrated untagged AMP; M, protein marker.

**Figure 5:**
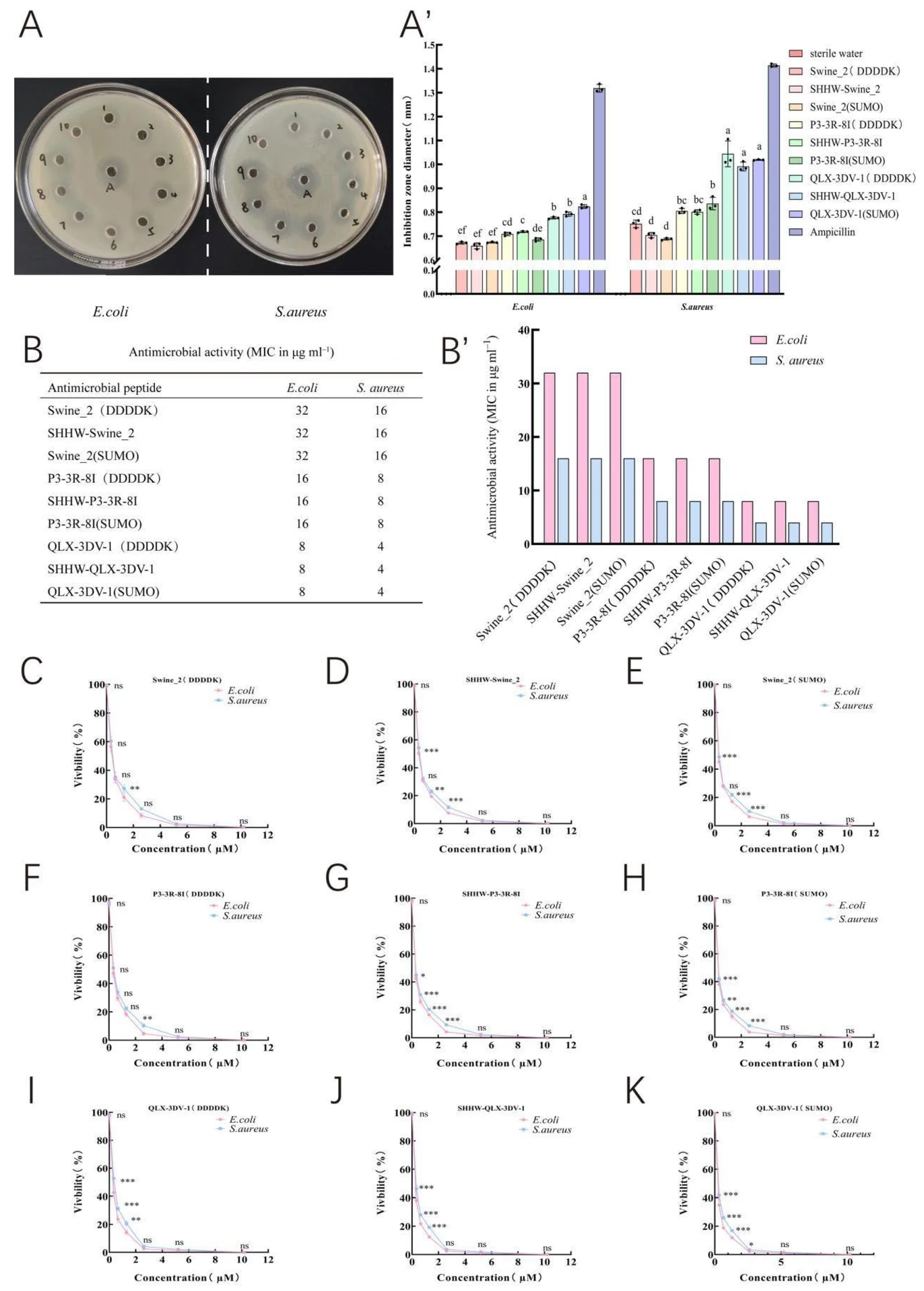
Antibacterial activity of the nine AMPs, assessed by agar well diffusion, MIC determination, and a bacterial viability assay. (**A**,**A′**) Representative inhibition zones against *E. coli* and *S. aureus* on LB agar plates; 50 μL (1 μg/μL) of each AMP was added per well. Numbers 1–9 correspond to Swine_2 (DDDDK) (1), SHHW-Swine_2 (2), Swine_2 (SUMO) (3), P3-3R-8I (DDDDK) (4), SHHW-P3-3R-8I (5), P3-3R-8I (SUMO) (6), QLX-3DV-1 (DDDDK) (7), SHHW-QLX-3DV-1 (8), and QLX-3DV-1 (SUMO) (9); number 10 is sterile water and A is ampicillin. Different lowercase letters (a, b, c, d, e, f) indicate significant differences among groups (*p* < 0.05). Groups sharing the same letter are not significantly different. (**B**,**B′**), The MIC values tabulated in this panel in the previous version have been transferred to Table 4. (**C**–**K**) Concentration-dependent decrease in the relative viability of *E. coli* and *S. aureus* for each of the nine AMP preparations; data points are the mean ± SD of three independent experiments, each performed in triplicate; and asterisks indicate significant differences among the three cleavage strategies for the same peptide backbone (one-way ANOVA with Tukey’s post-hoc test; ns indicates no significant difference, * *p* < 0.05, ** *p* < 0.01, *** *p* < 0.001).

**Table 1:** Profiles of the nine recombinant expression vectors, the corresponding AMPs, and the cleavage strategies.

No.	Vector	Fusion Tag System	Target AMP	Cleavage Method	Recognition Motif	Post-Cleavage AMP Name
1	pET32a	His-Trx-DDDDK-Swine_2	Swine_2	Enterokinase	DDDDK↓	Swine_2 (DDDDK)
2	pET32a	His-Trx-DDDDK-P3-3R-8I	P3-3R-8I	Enterokinase	DDDDK↓	P3-3R-8I (DDDDK)
3	pET32a	His-Trx-DDDDK-QLX-3DV-1	QLX-3DV-1	Enterokinase	DDDDK↓	QLX-3DV-1 (DDDDK)
4	pET32a	His-Trx-DDDDK-GSHHW-Swine_2	Swine_2	SNAC chemical cleavage	G↓SHHW	SHHW-Swine_2
5	pET32a	His-Trx-DDDDK-GSHHW-P3-3R-8I	P3-3R-8I	SNAC chemical cleavage	G↓SHHW	SHHW-P3-3R-8I
6	pET32a	His-Trx-DDDDK-GSHHW-QLX-3DV-1	QLX-3DV-1	SNAC chemical cleavage	G↓SHHW	SHHW-QLX-3DV-1
7	pET3C-SUMO	His-SUMO-Swine_2	Swine_2	SUMO protease	SUMO↓	Swine_2 (SUMO)
8	pET3C-SUMO	His-SUMO-P3-3R-8I	P3-3R-8I	SUMO protease	SUMO↓	P3-3R-8I (SUMO)
9	pET3C-SUMO	His-SUMO-QLX-3DV-1	QLX-3DV-1	SUMO protease	SUMO↓	QLX-3DV-1 (SUMO)

**Table 2:** Physicochemical parameters of the native and SNAC-tagged AMP variants.

Peptide	Sequence	Length	Net Charge	Hydrophobicity	Polar Residues (N/%)	Nonpolar Residues (N/%)
Swine_2	NKTKELNKKTARKNYKKIIKKIARKNYKKIIKKIARKNYKK	41	19	−1.70	31/75.6%	10/24.4%
GSHHW-Swine_2	GSHHWNKTKELNKKTARKNYKKIIKKIARKNYKKIIKKIARKNYKK	46	21	−1.55	34/73.9%	12/26.1%
P3-3R-8I	WNIIPIRRWNIIPIRHWNIIP	21	4	−0.17	10/47.6%	11/52.4%
GSHHW-P3-3R-8I	GSHHWWNIIPIRRWNIIPIRHWNIIP	26	6	−0.11	12/46.2%	14/53.8%
QLX-3DV-1	LKRFWRSPWKIKWK	14	6	−0.76	8/57.1%	6/42.9%
GSHHW-QLX-3DV-1	GSHHWLKRFWRSPWKIKWK	19	8	−0.63	10/52.6%	9/47.4%

**Table 3:** Recovery efficiency of the antimicrobial peptides, calculated relative to the theoretical maximum yield of the released peptide.

No.	Fusion Protein	MW of Fusion Protein (Da)	MW of Released Peptide (Da)	Protein Yield of Fusion Protein (μg)	Final Sample Volume (mL)	Measured Peptide Concentration † (mg/mL)	Measured Peptide Mass (μg)	Recovery (%)
1	His-Trx-DDDDK-Swine_2	20,258	5062	25.0	1.0	0.015	15.0	60.00%
2	His-Trx-DDDDK-P3-3R-8I	17,917	2721	15.2	1.0	0.0039	3.90	25.66%
3	His-Trx-DDDDK-QLX-3DV-1	17,156	1959	11.4	1.0	0.0038	3.80	33.33%
4	His-Trx-DDDDK-GSHHW-Swine_2	20,863	5610	26.9	1.0	0.0041	4.10	15.24%
5	His-Trx-DDDDK-GSHHW-P3-3R-8I	18,523	3269	17.6	1.0	0.0034	3.40	19.32%
6	His-Trx-DDDDK-GSHHW-QLX-3DV-1	17,761	2507	14.1	1.0	0.0044	4.40	31.21%
7	His-SUMO-Swine_2	17,208	5062	29.4	1.0	0.0047	4.70	15.99%
8	His-SUMO-P3-3R-8I	14,868	2721	18.3	1.0	0.0055	5.50	30.05%
9	His-SUMO-QLX-3DV-1	14,106	1959	13.9	1.0	0.0053	5.30	38.13%

**Table 4:** Minimum inhibitory concentrations of the nine AMP preparations against *E. coli* ATCC 8739 and *S. aureus* ATCC 25923.

AMP	Cleavage Strategy	MW (Da)	MIC, *E. coli* ATCC 8739 (μg/mL)	(μM)	MIC, *S. aureus* ATCC 25923 (μg/mL)	(μM)
Swine_2 (DDDDK)	Enterokinase	5062	32	6.3	16	3.2
SHHW-Swine_2	SNAC chemical cleavage	5610	32	5.7	16	2.9
Swine_2 (SUMO)	SUMO protease	5062	32	6.3	16	3.2
P3-3R-8I (DDDDK)	Enterokinase	2721	16	5.9	8	2.9
SHHW-P3-3R-8I	SNAC chemical cleavage	3269	16	4.9	8	2.4
P3-3R-8I (SUMO)	SUMO protease	2721	16	5.9	8	2.9
QLX-3DV-1 (DDDDK)	Enterokinase	1959	8	4.1	4	2.0
SHHW-QLX-3DV-1	SNAC chemical cleavage	2507	8	3.2	4	1.6
QLX-3DV-1 (SUMO)	SUMO protease	1959	8	4.1	4	2.0

## Data Availability

The original contributions presented in this study are included in the article/Supplementary Material. Further inquiries can be directed to the corresponding author.

## Supplementary Materials

The following supporting information can be downloaded at: https://www.mdpi.com/article/10.3390/cimb48100983/s1.
