# Supplementary material for: Comparative Analysis of Three Preparation Strategies for AntiMicrobial Peptides and Their Antibacterial Efficacy
Source: Curr Issues Mol Biol. 2026 Sep 24;48(10):983. doi: 10.3390/cimb48100983 (PMC13703638; doi:10.3390/cimb48100983)
Supplement: Supplementary file 1 [file cimb-48-00983-s001.zip › cimb-4554476-Supplementary.pdf]

Supplementary Table S1 Antimicrobial peptide sequences and primers

**Antimicrobial peptide**	**amino acid sequence (N→C)**	**deduceed gene sequence (5’→3’)**	**Primer(5’→3’)**
Swine_2	NKTKELNKKTA<br>RKNYKKIIKKIAR<br>KNYKKIIKKIAR<br>KNYKK	AATAAAACCAAAGAACTGAATAAAAAAA<br>CCGCGCGTAAAAATTATAAAAAAATTATT<br>AAAAAAATTGCGCGTAAAAATTATAAAAA<br>AATTATTAAAAAAATTGCGCGTAAAAATT<br>ATAAAAAA	P1: CGGGGTACCAATAAAACCAAAGAACTGAATA<br>P2: TATTCAGTTCTTTGTTTTATAATTTTTACGC<br>P3: CGCGGATCCTTGCGCGTAAAAATTATAAAAAA
GSHHW-Swine_2	GSHHWNKTKEL<br>NKKTARKNYKKI<br>IKKIARKNYKKII<br>KKIARKNYKK	GGCAGCCATCATTGGAATAAAACCAAAGA<br>ACTGAATAAAAAAACCGCGCGTAAAAATT<br>ATAAAAAAATTATTAAAAAAATTGCGCGT<br>AAAAATTATAAAAAAATTATTAAAAAAAT<br>TGCGCGTAAAAATTATAAAAAA	P1: CGGGGTACCGGCAGCCATCATTGGAATAAA<br>P2: TTTATTCCAATGATGGTTTTATAATTTTTACGC<br>P3: CGCGGATCCTTGCGCGTAAAAATTATAAAAAA
SUMO-Swine_2	GMSDSEVNQEA<br>KPEVKPEVKPET<br>HINLKVSDGSSEI<br>FFKIKKTTPLRRL<br>MEAFAKRQGKE<br>MDSLRFLYDGIRI<br>QADQTPEDLDM<br>EDNDIIEAHREQI<br>GGNKTKELNKK<br>TARKNYKKIIKKI<br>ARKNYKKIIKKI<br>ARKNYKK	GGCATGTCGGACTCAGAAGTCAATCAAGA<br>AGCTAAGCCAGAGGTCAAGCCAGAAGTC<br>AAGCCTGAGACTCACATCAATTTAAAGGT<br>GTCCGATGGATCTTCAGAGATCTTCTTCAA<br>GATCAAAAAGACCACTCCTTTAAGAAGGC<br>TGATGGAAGCGTTCGCTAAAAGACAGGGT<br>AAGGAAATGGACTCCTTAAGATTCTTGTA<br>CGACGGTATTAGAATTCAAGCTGATCAGA<br>CCCCTGAAGATTTGGACATGGAGGATAAC<br>GATATCATTGAGGCTCACAGAGAACAGAT<br>TGGTGGTAATAAAACCAAAGAACTGAATA<br>AAAAAACCGCGCGTAAAAATTATAAAAA<br>AATTATTAAAAAAATTGCGCGTAAAAATT<br>ATAAAAAAATTATTAAAAAAATTGCGCGT<br>AAAAATTATAAAAAA	SUMO P1：CTCCATATGGGCATGTCGGACTCAGAAGTC<br>SUMO P2：<br>CTGTTCTCTGTGAGCCTCCAGTTCTTTGGTTTTATT<br>Swine_2 P1：<br>AATAAAACCAAAGAACTGAATAAAAAAACCGCGCGT<br>Swine_2 P2：<br>CGCGTAAAAATTATAAAAAAGGATCCGCG
P3-3R-8I	WNIIPIRRWNIIPI<br>RHWNIIP	TGGAATATTATTCCGATTCGTCGTTGGAAT<br>ATTATTCCGATTCGTCATTGGAATATTATT<br>CCG	P1: CGGGGTACCTGGAATATTATTCCGATTCG<br>P2: CGAATCGGAATAATACGGAATAATATT<br>P3: CGCGGATCCCGGAATAATATTCCAAT
GSHHW-P3-3R-8I	GSHHWWNIIPIR<br>RWNIIPIRHWNII<br>P	GGCAGCCATCATTGGTGGAATATTATTCC<br>GATTCGTCGTTGGAATATTATTCCGATTCG<br>TCATTGGAATATTATTCCG	P1: CGGGGTACCGGCAGCCATCATTGGTGGA<br>P2: TCCACCAATGATGGCGGAATAATATT<br>P3: CGCGGATCCCGGAATAATATTCCAAT

SUMO-P3-3R-8I	GMSDSEVNQEAKPEVKPEVKPETHINLKVSDGSSEIFFKIKKTTPLRRLMEAFAKRQGKEMDSLRFLYDGIRIQADQTPEDLDMEDNDIIEAHREQIGGWNIIPIRRWNIIPIRHWNIIP	GGCATGTCGGACTCAGAAGTCAATCAAGAAGCTAAGCCAGAGGTCAAGCCAGAAGTCAAGCCTGAGACTCACATCAATTTAAAGGTGTCCGATGGATCTTCAGAGATCTTCTTCAAGATCAAAAAGACCACTCCTTTAAGAAGGCTGATGGAAGCGTTCGCTAAAAGACAGGGTAAGGAAATGGACTCCTTAAGATTCTTGTACGACGGTATTAGAATTCAAGCTGATCAGACCCCTGAAGATTTGGACATGGAGGATAACGATATCATTGAGGCTCACAGAGAACAGATTGGTGGTTGGAATATTATTCCGATTCGTCGTTGGAATATTATTCCGATTCGTCATTGGAATATTATTCCG	SUMO P1：CTCCATATGGGCATGTCGGACTCAGAAGTC<br>SUMO P2：CTGTTCTCTGTGAGCCTCAATCGGAATAATATTCCA<br>P3-3R-8I P1：TGGAATATTATTCCGATTCGTCGTTGGAATATT<br>P3-3R-8I P2：GACGAATCGGAATAATAAGGATCCGCG
QLX-3DV-1	LKRFWRSPWKIKWK	CTGAAACGTTTTTGGCGTAGCCCGTGGAAAATTAAATGGAAA	P1: CGGGGTACCCTGAAACGTTTTTGGCG<br>P2: CGCCAAAAACGTTTCCATTTAAT<br>P3: CGCGGATCCTTTCCATTTAATTTT
GSHHW-QLX-3DV-1	GSHHWLKRFWRSPWKIKWK	GGCAGCCATCATTGGCTGAAACGTTTTTGGCGTAGCCCGTGGAAAATTAAATGGAAA	P1: CGGGGTACCGGCAGCCATCATTGGCT<br>P2: AGCCAATGATGGTTTCCATTTAAT<br>P3: CGCGGATCCTTTCCATTTAATTTT
SUMO-QLX-3DV-1	GMSDSEVNQEAKPEVKPEVKPETHINLKVSDGSSEIFFKIKKTTPLRRLMEAFAKRQGKEMDSLRFLYDGIRIQADQTPEDLDMEDNDIIEAHREQIGGLKRFWRSPWKIKWK	GGCATGTCGGACTCAGAAGTCAATCAAGAAGCTAAGCCAGAGGTCAAGCCAGAAGTCAAGCCTGAGACTCACATCAATTTAAAGGTGTCCGATGGATCTTCAGAGATCTTCTTCAAGATCAAAAAGACCACTCCTTTAAGAAGGCTGATGGAAGCGTTCGCTAAAAGACAGGGTAAGGAAATGGACTCCTTAAGATTCTTGTACGACGGTATTAGAATTCAAGCTGATCAGACCCCTGAAGATTTGGACATGGAGGATAACGATATCATTGAGGCTCACAGAGAACAGATTGGTGGTCTGAAACGTTTTTGGCGTAGCCCGTGGAAAATTAAATGGAAA	SUMO P1：CTCCATATGGGCATGTCGGACTCAGAAGTC<br>SUMO P2：CTGTTCTCTGTGAGCCTCACGCCAAAAACGTTTCAG<br>QLX-3DV-1 P1：CTGAAACGTTTTTGGCGTAGCCCGTGGAAAAT<br>QLX-3DV-1 P2：TTTCCATTTAATTTTGGATCCGCG
